# Optimizing Volunteer Engagement and voluntary work design for Healthy Aging

**DOI:** 10.1093/geroni/igaf122.1948

**Published:** 2025-12-31

**Authors:** Shiyu Lu

**Affiliations:** The University of Hong Kong, Hong Kong, China (People’s Republic)

## Abstract

Volunteering is crucial for healthy aging, yet research has often prioritized volunteering quantity over role quality (e.g., psychological states where individuals are dedicated to their activities). This study investigates how volunteering engagement, one-year volunteering hours, and voluntary work design (VWD) influence subjective well-being (SWB) and cognition in older adults. The cross-sectional study involved 457 participants aged 55 and above in Hong Kong from 2023 to 2024. Volunteer engagement was evaluated using the adapted Chinese Utrecht Work Engagement Scale. The VWD Questionnaire assessed aspects like skill variety, task identity, feedback, and social support and recognition within the volunteer roles. Results from structural equation models indicated that volunteer engagement (β = 0.06, p = 0.007), recognition (β = 0.24, p < 0.001), task identity (β = 0.80, p = 0.029), and social support within volunteer roles (β = 1.35, p = 0.016) positively associate with SWB. Both volunteering engagement and hours positively affect cognition, with cognitive benefits of feedback (β = 0.12, p = 0.044) and skill variety (β = 0.10, p = 0.046) mediated by volunteering hours, and cognitive benefits of social support within volunteer roles (β = 0.17, p = 0.049) by volunteer engagement. Cognitive benefits of recognition are jointly mediated by the quantity and quality of volunteering (β = 0.032, p = 0.002). These findings underscore the importance of role engagement in late-life volunteering and pinpoint the key voluntary work characteristics (e.g., skill variety, task identity, feedback, and social support and recognition) to create supportive volunteer environment as part of social prescribing for healthy aging.

